# Enteric methane emissions and mitigation strategies within pasture-based dairy systems

**DOI:** 10.3168/jdsc.2025-0980

**Published:** 2026-04-20

**Authors:** Jane Kay, Konagh Garrett, Kirsty Verhoek, Lisanne Koning, Camila Muñoz, Cecile Martin, Ben Lahart

**Affiliations:** 1 DairyNZ, Hamilton, 3286, New Zealand; 2 Wageningen Livestock Research, Wageningen University & Research, 6700 AH Wageningen, the Netherlands; 3 Centro Regional de Investigación Remehue, Instituto de Investigaciones Agropecuarias. Osorno, 5290000, Chile; 4 UMR Herbivores, INRAE, VetAgro Sup, 63122 Saint-Genès-Champanelle, France; 5 Teagasc, Animal and Grassland Research and Innovation Centre, Moorepark, Fermoy, Co. Cork, P61 P302, Ireland

## Abstract

Enteric methane is the dominant GHG emitted from pasture-based dairy systems, defined for this review as dairy systems in which grazed pasture makes up the majority of feed intake. This review summarizes the main drivers of methane emissions and the mitigation options available in these systems. Evidence indicates that cows offered high-nutritive-value perennial ryegrass pasture, characterized by high OM digestibility and ME, and moderate NDF content, have lower methane conversion factors (CH_4_ as a percentage of gross energy intake) and methane yields (g CH_4_/kg DMI) than values currently used by the Intergovernmental Panel on Climate Change and national inventories. Potential methane mitigation strategies for these pasture-based systems fall into 3 broad categories: technological interventions (such as feed additives or vaccines), genetics, and nutritional approaches. Some mitigations show promise; however, their effectiveness under grazing conditions is often limited by delivery constraints, variable responses, and uncertainty around cost, persistence, and whole-farm impacts such as economics and GHG life cycle assessment outcomes. Overall, evidence indicates that well-managed pasture-based dairy systems have lower baseline methane emissions than current inventory estimates. However, beyond this lower baseline, no mitigation strategies have yet demonstrated cost-effective, scalable, and consistent emissions reductions across these systems.

## Highlights

• Baseline methane from pasture-based systems may be lower than inventory estimates.
• Baseline methane depends on cow intake levels and the nutritive value of the diet.
• Mitigation responses depend on the baseline methane and mode of action.
• Currently, there are no scalable or viable mitigations for pasture-based systems.

## Graphical Abstract

Baseline methane emissions from lactating cows grazing high-quality pasture may be lower than currently predicted by on-farm calculators and national inventories. Several mitigation strategies are being explored, including technological interventions, genetic selection, and nutritional approaches. However, the methane response to these strategies will depend on both the baseline emissions of the animals and the mode of action of the mitigation. Improving our understanding of baseline methane emissions, the factors that drive seasonal variation, and the responses to different mitigation strategies will support a more targeted approach to reducing methane emissions across the grazing season.

**Figure:**
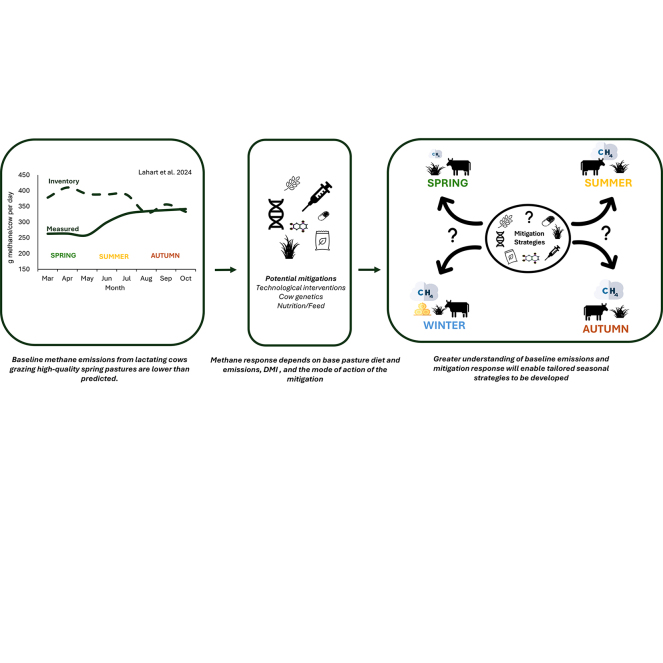

Enteric methane is the primary contributor to GHG emissions in pasture-based dairy systems (Rotz, 2018; Herron et al., 2022). Although these systems vary in feed management and production intensity, this review defines pasture-based systems as those in which grazed pasture is the main feed source, with supplementary feeds or crops used strategically at key times of the year. In this review, “pasture” refers to perennial ryegrass (**PRG**)-based swards, “crops” to deliberately sown forages such as brassicas or fodder beet, and “supplementary feed” to concentrates, silages and byproducts.

Such pasture-based systems are typical of New Zealand, Ireland, and parts of the European Union, Australia, and South America. In these regions, climate change is contributing to greater variability in pasture growth and seasonal feed supply and may therefore increase the use of alternative pasture species, crops, and supplementary feeds. In addition, dairy sectors within these regions face legislated and market-driven targets to reduce both absolute emissions (e.g., g CH_4_/ha) and emissions intensity (e.g., g CH_4_/kg fat- and protein-corrected milk [**FPCM**]; EEA, 2021; DAFM, 2022; Fonterra Co-operative Group, 2023; MfE, 2023; Ministerio del Medio Ambiente, 2025). Because profitability in these systems remains strongly linked to pasture and crop harvested per hectare (Dillon et al., 2005; Macdonald et al., 2008; Neal and Roche, 2019) methane mitigation strategies must reduce methane and total GHG emissions without compromising pasture and crop utilization or system resilience.

In pasture-based systems, baseline methane emissions and the response to mitigation vary with the basal diet, cow intake, stage of lactation, and the mitigation strategy (Hristov et al., 2015; Pickering et al., 2015; Eugène et al., 2021; Beauchemin et al., 2022), and thus should be evaluated under representative commercial farming scenarios. It is also important to integrate these data into whole-farm GHG life cycle assessment (**LCA**) frameworks to quantify the total GHG footprint for different systems and mitigations. This review summarizes current knowledge on baseline enteric methane emissions and mitigation options for pasture-based dairy systems. These are summarized in Table 1, which provides an overview of each strategy, its potential to reduce methane yield, optimal timing of application, mechanism of action, and key implementation challenges.

**Table 1:** Potential baseline methane emissions and mitigation strategies for pasture-based systems

Strategy	Baseline/mitigation	Potential reduction in methane yield	Best timing	Mechanism of action	Challenges
Baseline emissions	Spring, high-nutritive-value^1^ PRG pasture (lactating cow)	Baseline reduction (15%–20% lower than current Y_m_ or methane emission factor)	Spring, or potentially summer with irrigation	High OMD → faster passage rate → less fermentation per unit feed	Requires best-practice pasture management; seasonal variability
Technological interventions	Feed additives supplemented twice per % day (e.g., Bovaer, *Asparagopsis* sp., lipids)	Variable (generally less than when fed with a TMR)	Seasonal differences and farm systems fit^2^	Direct (e.g., blocks methyl-coenzyme M reductase), or indirect (limits hydrogen availability)	Delivery under grazing; regulatory approval; residue concerns, cost
	Bolus, vaccine, water trough	No published results		Slow-release rumen delivery, immune targeting of methanogens, more frequent delivery of inhibitor	Regulatory approval; residue concerns; cost
	Early-life programming	40% while Bovaer fed; 0% after treatment ceased	Birth to 12 wk	Inhibits methanogenesis (blocks methyl-coenzyme M reductase) during rumen colonization	No permanent effect when Bovaer offered to calves in pasture-based system
Genetics	High-genetic-merit cows/Jerseys and cross-bred cows	Small reduction in methane intensity (cumulative^3^)	Whole life	Breed animals with greater metabolic efficiency	Needs further validation with measurements across different systems
	Selected breeding (trait)	0.5%–1.0% per year in methane intensity (cumulative^3^)	Whole life	Altered rumen functionality and microbiome	Phenotyping under grazing; balancing breeding objectives; adoption speed
Nutrition	Alternative pasture species or crops (e.g., plantain or fodder beet)	Variable: can reduce methane yield, but often associated with greater methane production	Summer–winter	Inconsistent results: potentially (1) greater ME intake and milk production (2) altered rumen fermentation, (3) secondary compounds (e.g., tannins) reduce methanogens	Agronomic constraints; seasonal availability; variable efficacy; potential increases in total methane production
	Supplementary feeds (e.g., corn silage; high starch/energy concentrate)	Variable: can reduce methane yield when pasture quality is low	Summer–winter		Cost; substitution effects; LCA implications; efficacy varies with base diet, and type, amount, and timing of use of supplementary feed

1 ME 11.0–12.5 MJ/kg DM; OM digestibility >80%; NDF <45%.

2 Efficacy of feed additives with different pasture composition (e.g., spring vs. summer) requires further evaluation. For example, we postulate that the efficacy of transient inhibitors such as 3-NOP may be less in spring due to the lower rumen retention time of spring pasture, however, there is potential that the mitigation effect may be greater due to the high nutritive value of the spring pasture. How the use of supplementary feed fits in with the system needs to be considered.

3 May only be a small reduction in methane intensity (g CH_4_/kg FPCM) in the short term; however, in the long term, the reductions will be larger due to the cumulative effect.

Enteric methane production in ruminants is well characterized, with more than 50 years of research (Beauchemin et al., 2020; Khairunisa et al., 2023), and accurate quantification is essential to support evidence-based policy and mitigation strategies. However, large-scale, on-farm measurements of methane emissions remain logistically challenging, and national GHG inventories therefore rely on prediction models to estimate methane output. These models use standardized methane conversion factors (**Y_m_** values), which express methane as a percentage of gross energy intake (IPCC, 2019), or country-specific conversion factors, which express methane per unit of DMI (MPI, 2013).

To refine inventory factors, and evaluate mitigation strategies, methane output in pasture-based trials is quantified using several established measurement techniques. Each has methodological considerations that influence accuracy and comparability, particularly within pasture-based scenarios, and these must be considered when analyzing or interpreting data. A recent review by Hristov et al. (2025) summarized advances in methane measurement approaches, particularly for feed additive evaluation, and emphasized the importance of experimental design, diet context, and analytical consistency when interpreting methane data. We believe there are additional considerations when conducting trials in pasture-based dairy systems, and these are outlined briefly in the following paragraphs.

Respiration chambers provide precise, repeatable measurements over a short time frame through continuous monitoring of animal gas exchange; however, confinement can alter feeding, drinking, and rumination behavior, potentially yielding emission profiles that differ from those of grazing cows (Llonch et al., 2018; Zhao et al., 2020). The sulfur hexafluoride (SF_6_) tracer technique enables measurements under grazing conditions, providing daily emission estimates, but accuracy depends on careful characterization of SF_6_ release rate from each permeation tube because variation in release rate directly affects calculated emissions (Deighton et al., 2014). GreenFeed (**GF**; C-Lock Inc.) systems, widely used in housed and grazing contexts, collect repeated, short-duration flux measurements during voluntary or scheduled visits. With appropriate management and analyses, GF estimates align with chamber and SF_6_ results (Hammond et al., 2016; Velazco et al., 2016; Jonker and Waghorn, 2020). In grazing or zero-grazed studies, however, robust estimates require sufficient GF visit frequency, temporal distribution across the day, and analytical windows that align with the mitigation being evaluated. Methane emissions from grazing cows typically follow a bimodal daily pattern, with a smaller peak-to-trough ratio than from cows in housed TMR systems; however, the timing and magnitude of emissions can be more variable over time, driven by fluctuations in pasture composition, grazing allocation, and feeding behavior (Dressler et al., 2024). Therefore, GF data from pasture-based systems require aggregation windows long enough to capture sufficient animal visit numbers and diurnal coverage, but short enough to minimize confounding factors, such as changes in pasture composition, intake, and lactation stage, Recent pasture-based studies have applied defensible approaches to improve 24-h representation, including time-of-day binning of visits, mixed-model adjustment for visit time and other covariates, and sequential averaging across hour and week to derive more robust arithmetic means (Lahart et al., 2024; Dwan et al., 2025; Garrett et al., 2025). These studies indicate that in pasture-based systems, emissions can be accurately estimated over pasture-relevant periods, such as 14 d in grazing cows (Lahart et al., 2024; Dwan et al., 2025), and shorter periods, such as 7 d, in zero-grazed systems when daily visitation frequency is greater (e.g., Garrett et al., 2025).

Collectively, these indicate that respiration chambers, SF_6_ tracer, and GF systems can all provide scientifically valid estimates of methane emissions in pasture-based systems, but they also highlight the need for an accepted pasture-specific framework for methane data processing and interpretation. Additionally, when comparing methane data across experiments or conducting meta-analyses, consideration must be given to how DMI was measured or estimated. This is particularly important for methane yield and Y_m_, both of which depend directly on DMI. Although milk production is readily measured and used to express methane intensity, DMI remains difficult to estimate in grazing cows. Consequently, some experiments use zero-grazed conditions to allow more controlled intake measurement, whereas others estimate intake under grazing conditions using approaches such as n-alkanes or energetic back calculations (e.g., Lahart et al., 2024; Dwan et al., 2025; Kar-Klootwijk et al., 2026). These differences in intake methodology should be considered alongside methane measurement techniques and analyses when interpreting methane data across experiments.

An increasing body of literature on methane emissions from pasture-based systems supports refinement of current Y_m_ and methane yield values. Because methane production is influenced by diet and feeding systems, studies should be interpreted relative to conditions representative of well-managed grazing systems for the season evaluated. Accordingly, baseline Y_m_ and methane yield values should be derived from conditions representative of industry practice, including pre- and postgrazing mass, sward structure, botanical composition, nutritive value and DMI (Muñoz et al., 2016; Eugène et al., 2021; Loza et al., 2021; Lahart et al., 2024; Kar-Klootwijk et al., 2026). Studies in which pasture characteristics or intake are not representative of the season or system of interest (e.g., a spring diet for lactating cows with low ME and organic matter digestibility [**OMD**], or high NDF) should be interpreted with caution when establishing baseline emission values, or benchmarking mitigation effects.

Lactating cows fed pasture of high nutritive value characterized by high ME (e.g., >11.5 MJ/kg DM), high OMD (e.g., OMD >80%), and moderate NDF (e.g., NDF <40%) produce methane yields that are ~15% to 20% lower than predicted using default Y_m_ and county-specific conversion factors. For example, in Ireland, Lahart et al. (2024) reported a measured Y_m_ of 0.046 for cows grazing high-nutritive value spring pasture, compared with the default value of 0.063 (IPCC, 2019). Similarly, in New Zealand and the Netherlands, methane yields of 17.5 and 15.5 g CH_4_/kg DMI, respectively, were measured when cows consumed high-nutritive-value spring pasture (Dalton et al., 2025; Kar-Klootwijk et al., 2026) compared with a default value of 21.6 g CH_4_/kg DMI (MPI, 2013). Coupled with peak milk production in spring-calving systems, these lower methane yields translate into lower measured methane intensity (g CH_4_/kg product) during this period than currently predicted using inventory models with default values.

In addition, inventory model outputs predict that cows with higher genetic merit (based on national breeding indexes) and greater milk production may have lower methane intensity (i.e., g CH_4_/kg FPCM) but higher total methane emissions because of an assumed increase in DMI (Zhang et al., 2019; Lahart et al., 2021). However, direct methane measurements indicate that although high-genetic-merit cows, defined as the top 1% within the Irish Economic Breeding Index, produced more milk, they did not exhibit the associated increase in total methane emissions predicted by inventory models (Lahart et al., 2024). These results indicate that inventory models assuming uniform efficiency across animals differing in genetic merit may also overestimate methane emissions from elite animals.

Understanding variation in methane emissions in pasture-based systems is critical not only for establishing accurate Y_m_ values, but also for designing effective mitigation strategies. This is because the response to a given mitigation depends on several factors, including baseline methane emissions, the mechanism diving these, and the mode of action of the mitigation itself. Accordingly, mitigation potential is unlikely to be uniform across pasture-based systems or across seasons due to changes in pasture characteristics and intake (Jacobs et al., 1999). For example, although some evidence suggests that diets of high-nutritive value may provide greater scope for methane reduction with products such as 3-nitrooxypropanol (**3-NOP**; van Gastelen et al., 2022), the efficacy of such transient inhibitors may be affected by rumen kinetics and retention time, meaning responses may differ in pasture-based systems (Costigan et al., 2024). Practical feasibility should also be considered, as some strategies may be easier and more cost effective to implement when supplementary feeds are more typically used, such as during the shoulders of the season or periods of feed deficit. Because the relationship between methane emissions and mitigation responses is multifactorial, further research is required to determine how interactions among the basal diet, animal physiology, and mitigation type influence methane responses across seasons in pasture-based systems.

Within this seasonal and dietary context, methane mitigation strategies in pasture-based systems can be broadly grouped into 3 categories: technological interventions, cow genetics, and nutritional or feed approaches. Technological interventions include discrete mitigations such as feed additives, vaccines, or other methane-inhibiting compounds that directly reduce methanogenesis or modify rumen fermentation. Genetic strategies include selection for animals with greater efficiency or lower methane yields, whereas nutritional or feed approaches include changes to pasture species, crops, and supplementary feeds. Although several reviews of methane mitigation have focused on dairy cows fed TMR in housed systems (Hristov et al., 2013; Beauchemin et al., 2020, 2022), reviews focused specifically on pasture-based dairy systems remain limited (Pacheco et al., 2014; Vargas et al., 2022; Kalehe Kankanamge et al., 2025).

Regarding technological interventions in pasture-based systems, feed additives or methane inhibitors remain an option. Among natural methane inhibitors, the red seaweed *Asparagopsis* spp. is the most potent because of its high bromoform content, which inhibits methyl-coenzyme M reductase in the methanogenesis pathway (Chagas et al., 2019). In TMR-fed cows, *Asparagopsis* spp. reduced methane by 40% to 90% (Kinley et al., 2020), but in grazing cattle, efficacy is typically lower at ~20% to 40% (Alvarez-Hess et al., 2023; C. Muñoz, Centro Regional de Investigación Remehue, Instituto de Investigaciones Agropecuarias, Osorno, Chile, unpublished data), largely due to infrequent doses offered twice daily at milking times. Additional concerns with red seaweed include palatability, variability in bromoform concentration, potential residues in milk and meat, and potential loss of efficacy over time (Ahmad et al., 2025; Angellotti et al., 2025).

Among synthetic methane inhibitors, 3-NOP (Bovaer, Elanco) is one of the most extensively researched and used in housed TMR systems globally, where it typically achieves methane reductions of 20% to 40% (Hristov et al., 2015; Dijkstra et al., 2018). In grazing systems, however, its short ruminal half-life (~3 h) combined with twice-daily delivery (linked to standard milking frequency) limits the efficacy to 5% to 10% (Costigan et al., 2024; Muñoz et al., 2024). To address this delivery challenge, alternative routes are being explored. For example, administration of 3-NOP via drinking water may provide more continuous uptake (Rivelli et al., 2025), although cow drinking behavior varies with diet, weather, and stage of lactation, potentially affecting dose consistency, both between animals and throughout the year (Jago et al., 2005). In addition, delivery of methane inhibitors via open or exposed water systems may reduce effectiveness due to degradation or volatilization, light exposure, temperature, or prolonged residence time in troughs. Together, these factors increase uncertainty around actual inhibitor intake, make it more difficult to remain within regulatory guidelines, and may contribute to inconsistent methane mitigation responses under grazing conditions.

More broadly, delivery constraints in pasture-based systems have driven interest in alternative mechanisms designed to provide a more sustained mode of action. One such approach is synthetic bromoform, the active compound in red seaweed, delivered via a bolus or offered as a slow-release formulation (MPI, 2025), although efficacy data are currently unpublished and food safety and regulatory barriers remain unresolved. Vaccines targeting methanogens are also being developed and align well with pasture-based systems, as they are already routinely used and do not require changes to the farm system; however, efficacy results to date have been small and variable (Wedlock et al., 2013). Early-life intervention from birth to weaning is an alternative approach, based on the concept of altering rumen microbial colonization and development during a period of greater plasticity (Abecia et al., 2013; Meale et al., 2021). However, recent data indicate no permanent long-term efficacy in pasture-based systems (Kay et al., 2024). Overall, methane-reducing compounds delivered via different mechanisms hold potential for pasture-based systems; however, consistent delivery, efficacy, regulatory approval, and cost remain challenging; thus, as yet, there are no technological interventions that can achieve meaningful, viable, and durable methane reductions at scale.

Altering genetics (e.g., through specific breeding traits or breeding indexes) offers a different mitigation pathway with potentially permanent, and cumulative effects. As individual variation, and heritability has been reported in sheep and cattle, direct selection for low methane–emitting animals is plausible (Pinares-Patiño et al., 2013; Goopy et al., 2015; Rowe et al., 2019; Manzanilla-Pech et al., 2021; Smith et al., 2021; van Breukelen et al., 2022) and could deliver annual reductions of 0.5% to 1.0% (Pickering et al., 2015). In pasture-based systems, however, national breeding objectives and economic indexes already target improved animal productivity and efficiency, which contribute indirectly to methane mitigation. Therefore, any methane trait added to a breeding index would need to be balanced against existing breeding objectives to avoid compromising gains in productivity, efficiency, and economic performance.

Breed selection may also offer potential to influence methane emissions in pasture-based systems. Jersey cows typically emit less total methane per day than Holstein-Friesian (**HF**) cows due to their lower BW and maintenance requirements (Münger and Kreuzer, 2006; Thackaberry et al., 2010; Lahart et al., 2024). However, when expressed as methane yield, Jersey and HF cows have similar emissions. Given their greater milk fat and protein concentration and potential greater production efficiency (kg FPCM/kg DMI), Jersey cows might be expected to have lower methane intensity (g CH_4_/kg FPCM); however, results in the literature are inconsistent, potentially confounded by differences in cow genetic merit and feeding level.

Cross-bred cows (Jersey × HF), which are widely used in New Zealand's pasture-based systems to increase efficiency and profitability (Lopez-Villalobos et al., 2021), may also differ from HF cows in methane traits. Although data remain limited, recent evidence indicates lower methane intensity (CH_4_/kg FPCM) in cross-bred cows relative to HF cows, likely due to greater feed conversion efficiency with similar methane yield (Dwan et al., 2026). Given the prevalence of cross-bred genetics in New Zealand (LIC and DairyNZ, 2023), further research is required to determine whether breed could contribute to methane mitigation in pasture-based systems.

The third category of mitigations compatible with grazing systems is nutritional or feed-based strategies. Although not considered a mitigation per se, methane yield is typically lower (~20%) when lactating cows graze pasture of high nutritive value (often typical of well-managed spring pasture) compared with when they graze more mature, lower-nutritive-value pasture (Robertson and Waghorn, 2002; Muñoz et al., 2016; Loza et al., 2021; Lahart et al., 2024; Kar-Klootwijk et al., 2026). Several mechanisms have been proposed, including differences in pasture digestibility, fiber concentration, DMI, and rumen retention time. However, interpretation is challenging because these factors often change together, particularly when comparing across experiments. In particular, higher OMD is frequently associated with greater DMI, making it difficult to separate their independent effects. Lower methane yield associated with greater DMI is generally attributed to reduced rumen retention time, which limits the extent of fermentation per unit of feed (Johnson and Johnson, 1995; Pinares-Patiño et al., 2007; Hammond et al., 2014). By contrast, lower methane yield associated with greater OMD has been attributed to faster ruminal fermentation, and shifts in pathways that reduce acetate and butyrate production, thereby limiting substrate availability for methanogenesis. Overall, the available evidence indicates that methane yield declines as DMI increases, although few studies have evaluated this while holding diet composition constant. In a recent study, lactating and nonlactating cows consumed different amounts of the same zero-grazed pasture (~20 and ~10 kg DMI, respectively). Despite consuming pasture of the same nutritive value, cows with a greater intake had a 30% lower methane yield. This supports the theory that DMI is a key driver of methane yield (J. Dalton, DairyNZ, Hamilton, New Zealand, unpublished data)

In contrast, the relationship between OMD and methane emissions is less consistent. Although most studies report lower methane yields from cows offered pasture with greater OMD (Wims et al., 2010; Muñoz et al., 2016; Lahart et al., 2024; Dalton et al., 2025; Kar-Klootwijk et al., 2026), others have reported the opposite and attributed this to less fermentable substrate per unit of feed consumed (Pacheco et al., 2014; Della Rosa et al., 2022). These divergent results indicate that diet OMD alone does not explain variation in methane emissions within pasture-based systems, and that its effect is linked to intake level. This interpretation is consistent with Blaxter and Clapperton (1965), who established that in sheep, the relationship between OMD and methane yield was positive at maintenance intake, but became negative at higher intake levels.

One rumen-related factor that may also contribute to variation in methane emissions is pH, although its role in grazing systems remains uncertain. Lower mean rumen pH (~5.8–6.2), often associated with higher intakes of high-OMD pasture, has been linked with lower methane yield. However, the causal relationship remains unclear because pH dynamics in pasture-based systems differ from those in starch-rich TMR diets, where lactic acid accumulation is a major driver of low pH (Kolver and De Veth, 2002; McAuliffe et al., 2022). Other dietary factors, such as DM concentration and nitrate content, may also affect methane production, as may grazing behavior traits such as bite size, grazing intensity, and their associated effects on rumen dynamics (Rowe et al., 2019). More research is required to determine how pasture characteristics, DMI, rumen pH, and their interactions alter methane emissions and the identification of low-methane diets for pasture-based dairy cows.

Beyond variation in nutritive value and DMI within pasture-based systems, altering sward botanical composition may also influence methane emissions. Increasing climate variability and environmental pressures are driving greater botanical diversity in pasture-based systems, including the use of legumes, herbs, multispecies swards, and crops within grazing rotations. Inclusion of legumes such as clover in PRG pasture generally increases diet nutritive value (e.g., ME and OMD), DMI, and animal performance; however, effects on methane emissions are inconsistent (Lee et al., 2004; Dwan et al., 2025). Similarly, herbs such as plantain are attractive for nitrogen leaching mitigation, but methane responses are variable (Della Rosa et al., 2022; Dwan et al., 2026; Minogue et al., 2025). Across studies, methane reductions with plantain range from 0% to 28%; although the mechanisms are difficult to isolate because of variation in plantain inclusion level (30% to 100%), growth stage (e.g., vegetative vs. reproductive), cow stage of lactation and intake, and the base diet against which it is compared. Birdsfoot trefoil may also reduce methane through plant secondary compounds (Woodward et al., 2004), but similar to plantain, results vary depending on diet inclusion and other confounding factors. Brassica crops such as kale and fodder beet can also reduce methane when included at high dietary levels, but these inclusion rates carry animal health risks and are generally only practical for short periods (Sun et al., 2015).

Finally, genetically modified forages, such as high-lipid and high-ME PRG, and white clover with elevated condensed tannin concentrations, represent a promising concept for grazing systems (Beechey-Gradwell et al., 2020; Roldan et al., 2022); however, current regulatory constraints around genetic engineering limit research progress and potential uptake in some countries, including New Zealand. Research is also underway using non-genetically modified approaches to improve pasture traits such as digestibility or carbohydrate and fiber fractions (e.g., reduced lignin, or higher sugar content). These approaches may offer the potential to lower methane emissions, but they require further agronomic and animal evaluation to determine their performance within farm systems.

Beyond modifying pasture composition and forage type, methane emissions in grazing systems may also be influenced by the strategic use of supplementary feeds. In pasture-based systems, supplementary feeds such as concentrates, byproducts, and silages are typically used when feed supply does not meet feed demand, for example under high stocking rates or during periods of poor pasture growth. However, methane responses are highly variable and depend not only on supplement type and amount, but also on the characteristics of the basal pasture diet and the extent to which supplementation increases total DMI (Jiao et al., 2014; Pacheco et al., 2014; Soder and Brito, 2023; Della Rosa et al., 2025a,b; Garrett et al., 2025).

Among concentrates, starch-rich supplements can reduce methane yield when fed with lower ME and higher NDF pasture-based diets and when supplementation increases total DMI sufficiently to alter both rumen fermentation and rumen retention time (Della Rosa et al., 2025a). In contrast, when cows were offered a more representative high-quality spring pasture, Garrett et al. (2025) reported no change in methane yield or intensity, and daily methane production tended to increase, consistent with high pasture substitution and only a small change in diet composition and total DMI. Similar findings were reported by Lovett et al. (2005) and Muñoz et al. (2015), where starch-based concentrate supplementation increased daily methane production but did not significantly alter methane yield or intensity in cows grazing high-nutritive-value pasture. Recent work comparing high-starch, high-fiber, and mixed starch and fiber concentrates also indicates that differences among concentrate types in grazing systems may be driven as much by their effects on substitution and total DMI as by carbohydrate class per se. In late-lactation cows, Della Rosa et al. (2025b) reported that methane yield was unchanged across the concentrate types, but methane intensity tended to decline when the mixed concentrate resulted in greater DMI and milk production.

Byproducts and lipid-rich supplements provide a mechanistically different pathway for methane mitigation. Lipids may reduce methane through direct inhibition of methanogens and protozoa, reduced fiber digestion, and diversion of hydrogen toward biohydrogenation of UFA (McAllister et al., 1996; Beauchemin et al., 2008; Grainger and Beauchemin, 2011; Hristov et al., 2013). Meta-analyses in largely confined systems indicate that methane yield declines by ~0.66 to 1.0 g CH_4_/kg DMI, or by ~3% to 4%, for each 1% increase in dietary fat, although responses depend strongly on fatty acid profile and are generally more pronounced with unsaturated long-chain fatty acids (Patra and Saxena, 2010; Moate et al., 2011; Knapp et al., 2014). Under grazing conditions, however, responses are inconsistent and appear to depend on fatty acid source, dose, treatment duration, basal pasture composition, and method of delivery. For example, Pinares-Patiño et al. (2016) reported an 18% reduction in methane yield when canola oil was sprayed onto pasture for beef steers, whereas Muñoz et al. (2024) reported a transient 14% reduction in methane yield when whole cottonseed was fed twice daily to spring-grazing dairy cows, but no response to similar amounts of rapeseed or linseed. In contrast, Boland et al. (2020) reported methane reduction when linseed oil, but not soy oil, was fed to grazing dairy cows. These responses again appear to reflect both diet composition and intake dynamics, as lipid supplementation may alter DMI and pasture substitution as well as rumen methanogenesis. For commonly used byproducts such as palm kernel expeller, direct methane data under grazing conditions remain limited, and their mitigation potential should therefore still be considered uncertain.

By contrast, ensiled feeds are generally not a methane mitigation strategy in their own right. Relative to fresh pasture, pasture silage commonly increases dietary fiber concentration and lowers digestibility and therefore tends to increase methane yield and often methane intensity (Kennedy et al., 2026). Responses are not uniform, however, because silage type, fermentation quality, and inclusion level can all alter methane production. For example, maize silage may elicit a different response from pasture silage because of its greater starch concentration and lower fiber content, and methane outcomes will also depend on whether silage supplementation increases total DMI (Hammond et al., 2016; van Gastelen et al., 2019, 2022). Nevertheless, in grazing systems, ensiled forages are not generally considered a mitigation, but they should still be treated as distinct from fresh pasture when defining or interpreting methane data and should not be grouped with or treated as part of the baseline pasture diet.

Collectively, the methane response to supplementary feeds in grazing dairy systems is governed primarily by interactions among supplement composition, basal pasture quality, pasture substitution, and total DMI. This means supplementary feeds cannot be assumed to be inherently mitigative. Rather, their mitigation potential depends on whether they meaningfully alter intake and rumen function relative to the pasture they replace, and this must be evaluated across methane yield, total methane production, and methane intensity, as well as total GHG emissions within a whole-system context (Beauchemin et al., 2022; Soder and Brito, 2023; Della Rosa et al., 2025a,b; Garrett et al., 2025).

Overall, no single methane mitigation currently offers a scalable solution for pasture-based systems. Efficient grazing management and maintenance of pasture nutritive value may help reduce basal methane emissions, and an integrated strategy combining technological interventions, genetics, and nutritional approaches may provide incremental benefits. Stacking mitigations with different modes of action may offers further potential, though this requires careful in vivo testing to confirm whether effects are additive. Robust on-farm recording and verification of mitigation strategies will also be essential to ensure farmers receive appropriate recognition and credit.

In conclusion, pasture-based systems remain central to dairy production in temperate climates, where profitability is linked to pasture utilization. Enteric methane is the dominant source of GHG emissions on these farms, and current inventory methodologies appear to overestimate methane emissions from cows grazing well-managed pasture. Variation in methane emissions related to DMI, feed characteristics, and rumen dynamics reinforces the importance of trial design, representative basal pasture diets, measurement methods and analytical approaches when establishing baseline methane metrics and evaluating mitigation strategies for specific regions, systems, and seasons.

A range of technological interventions, genetic approaches, and feed-based strategies have been evaluated, although each faces challenges related to delivery, consistency, and scalability under grazing conditions. Mitigation approaches must therefore be assessed holistically so that gains in methane reduction do not come at the expense of farm profitability, other environmental outcomes (e.g., nitrogen efficiency), or total GHG emissions calculated using an LCA approach. They must also remain adaptive to increasing climate variability, which is likely to influence pasture growth, seasonal feed supply, and the use of diverse pasture species, crops, and supplementary feeds. Continued international collaboration, particularly between countries with pasture-based systems, will be critical to accelerate progress, harmonize methodologies, and develop emission profiles and mitigation strategies that are practical, effective, and sustainable.
